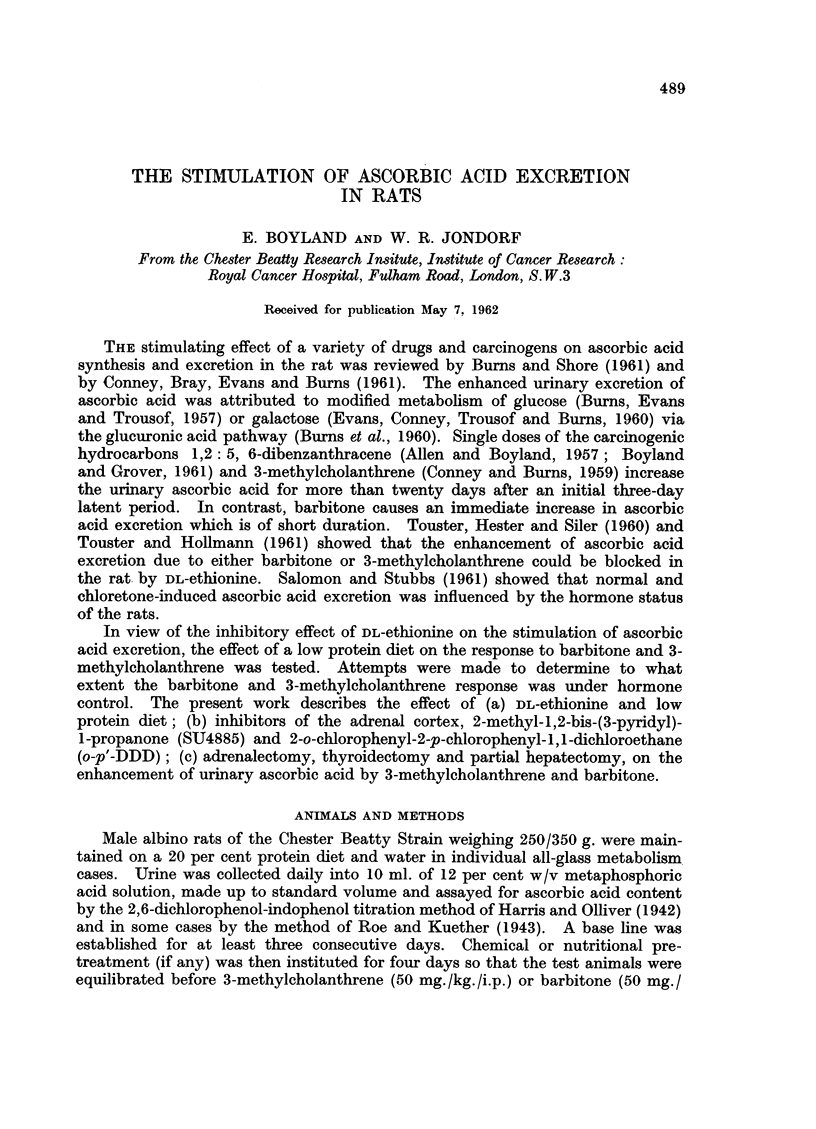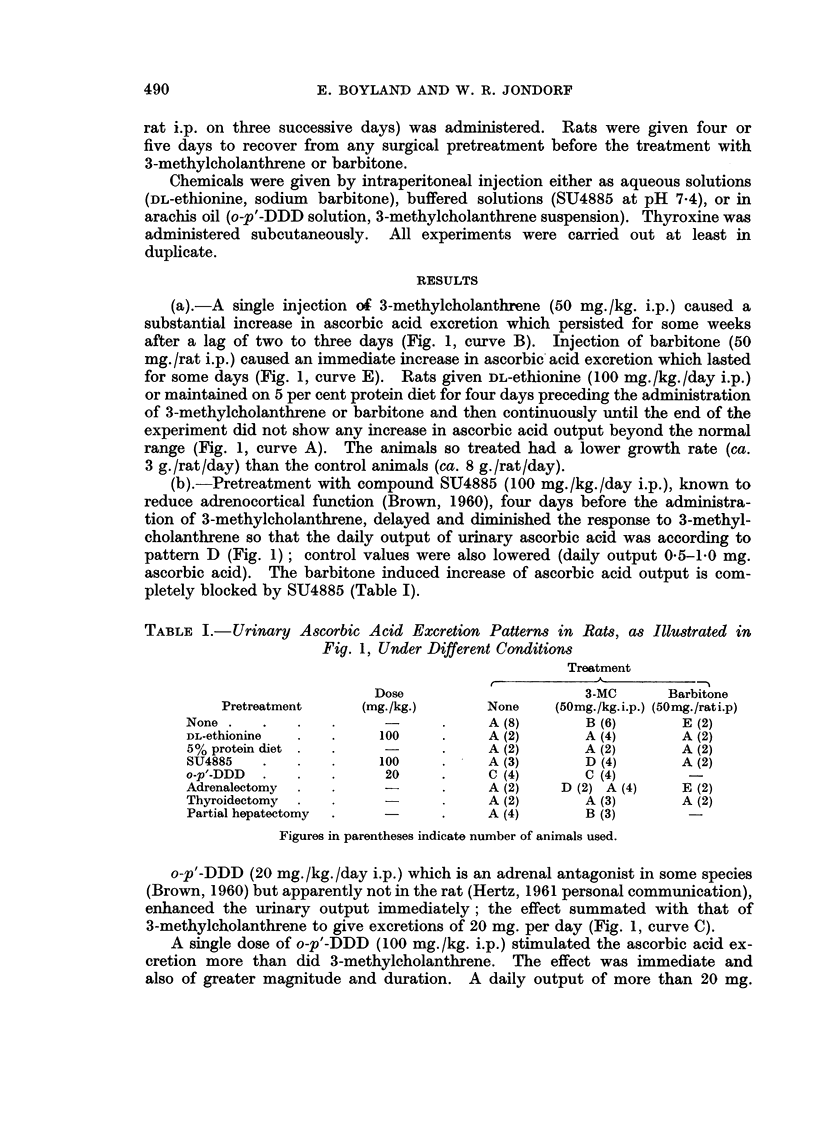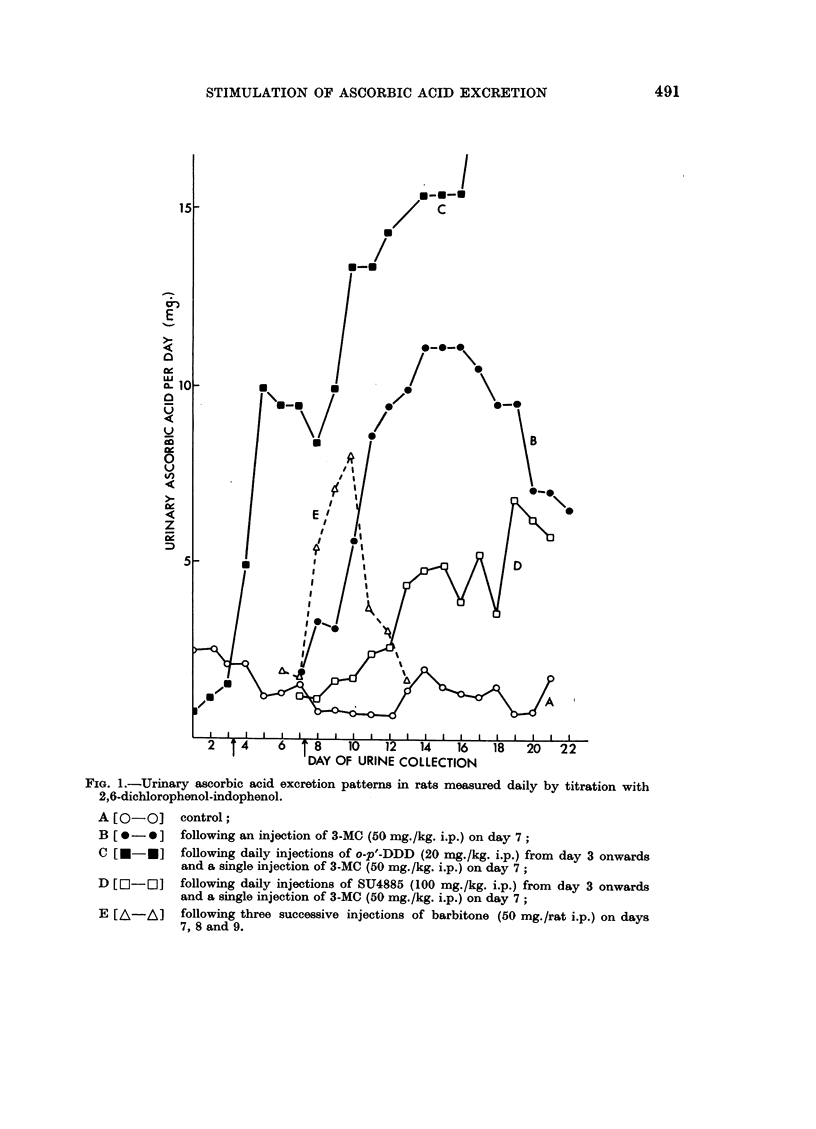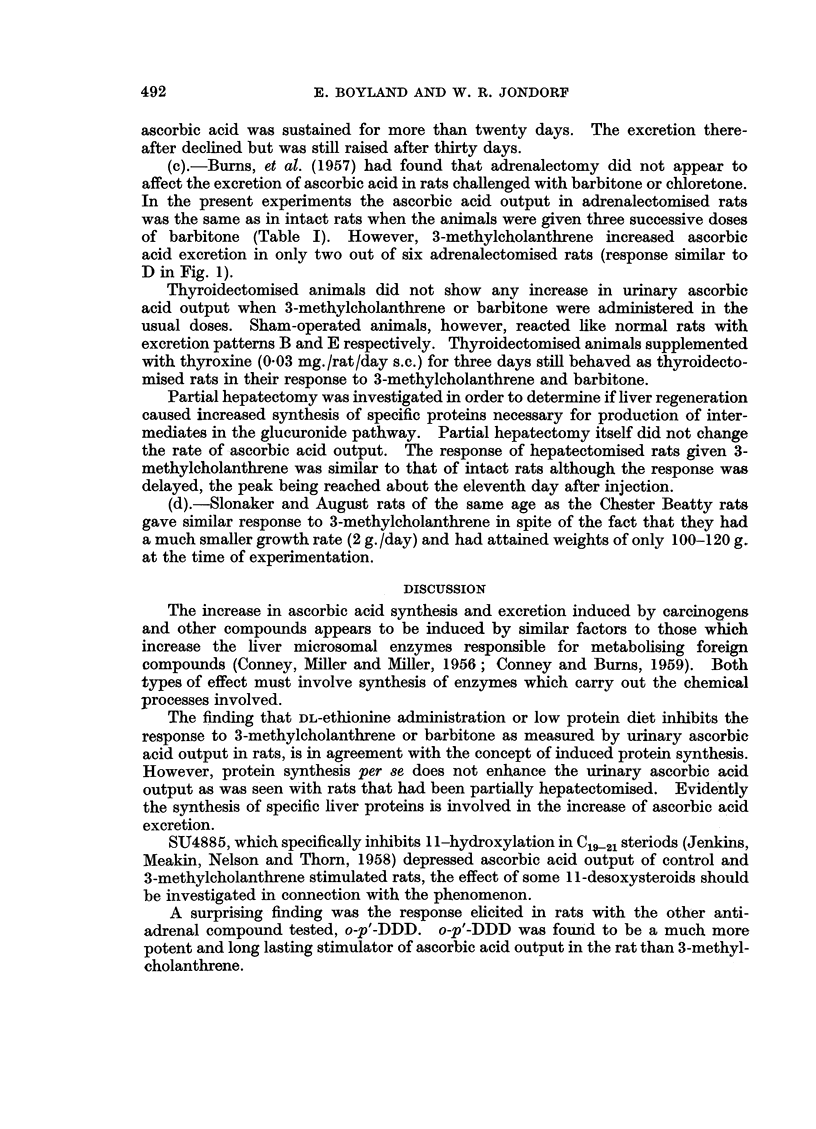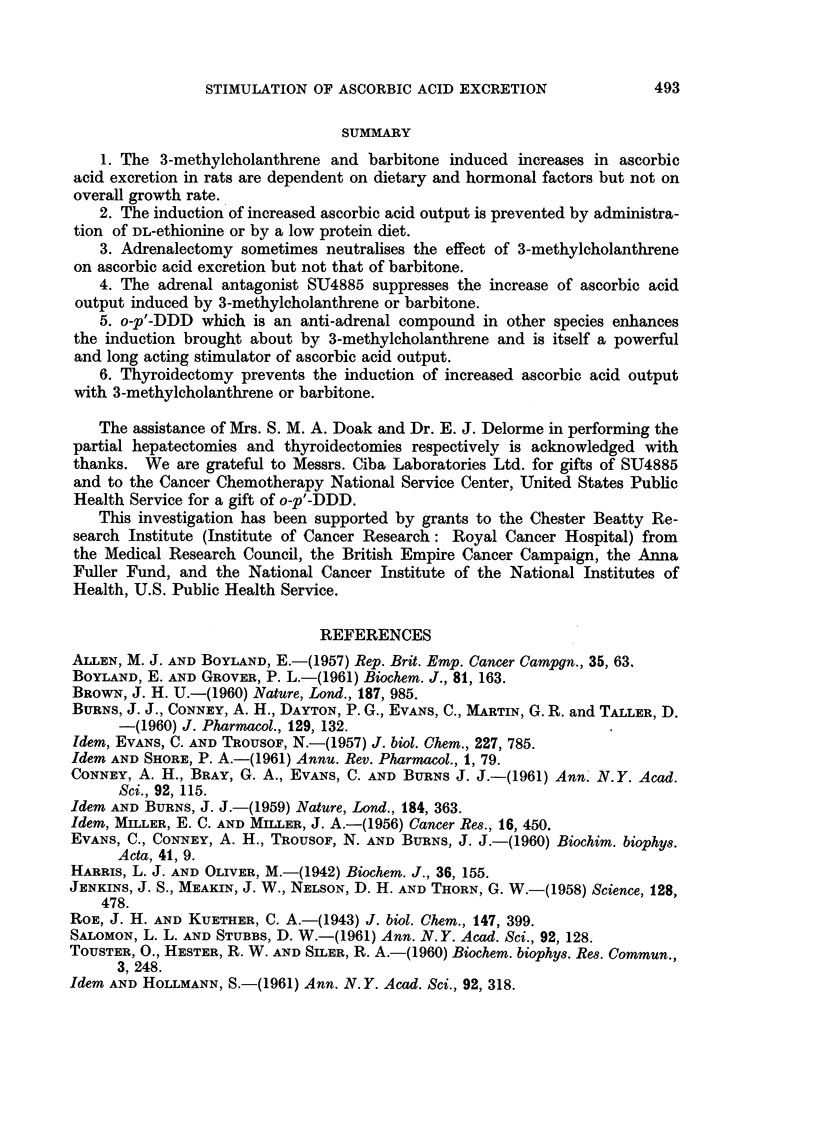# The Stimulation of Ascorbic Acid Excretion in Rats

**DOI:** 10.1038/bjc.1962.56

**Published:** 1962-09

**Authors:** E. Boyland, W. R. Jondorf


### THE STIMULATION OF ASCORBIC ACID EXCRETION IN RATS

E. BOYLAND AND W. R. JONDORF

*From the Chester Beatty Research Insitute, Institute of Cancer Research : Royal Cancer Hospital, Fulham Road, London, S.W.3*

Received for publication May 7, 1962

THE stimulating effect of a variety of drugs and carcinogens on ascorbic acid synthesis and excretion in the rat was reviewed by Burns and Shore (1961) and by Conney, Bray, Evans and Burns (1961). The enhanced urinary excretion of ascorbic acid was attributed to modified metabolism of glucose (Burns, Evans and Trousof, 1957) or galactose (Evans, Conney, Trousof and Burns, 1960) via the glucuronic acid pathway (Burns *et al.*, 1960). Single doses of the carcinogenic hydrocarbons 1,2 : 5, 6-dibenzanthracene (Allen and Boyland, 1957 ; Boyland and Grover, 1961) and 3-methylcholanthrene (Conney and Burns, 1959) increase the urinary ascorbic acid for more than twenty days after an initial three-day latent period. In contrast, barbitone causes an immediate increase in ascorbic acid excretion which is of short duration. Touster, Hester and Siler (1960) and Touster and Hollmann (1961) showed that the enhancement of ascorbic acid excretion due to either barbitone or 3-methylcholanthrene could be blocked in the rat by DL-ethionine. Salomon and Stubbs (1961) showed that normal and chloretone-induced ascorbic acid excretion was influenced by the hormone status of the rats.

In view of the inhibitory effect of DL-ethionine on the stimulation of ascorbic acid excretion, the effect of a low protein diet on the response to barbitone and 3-methylcholanthrene was tested. Attempts were made to determine to what extent the barbitone and 3-methylcholanthrene response was under hormone control. The present work describes the effect of (a) DL-ethionine and low protein diet ; (b) inhibitors of the adrenal cortex, 2-methyl-1,2-bis-(3-pyridyl)-1-propanone (SU4885) and 2-*o*-chlorophenyl-2-*p*-chlorophenyl-1,1-dichloroethane (*o*-*p*'-DDD) ; (c) adrenalectomy, thyroidectomy and partial hepatectomy, on the enhancement of urinary ascorbic acid by 3-methylcholanthrene and barbitone.

### ANIMALS AND METHODS

Male albino rats of the Chester Beatty Strain weighing 250/350 g. were maintained on a 20 per cent protein diet and water in individual all-glass metabolism cases. Urine was collected daily into 10 ml. of 12 per cent w/v metaphosphoric acid solution, made up to standard volume and assayed for ascorbic acid content by the 2,6-dichlorophenol-indophenol titration method of Harris and Olliver (1942) and in some cases by the method of Roe and Kuether (1943). A base line was established for at least three consecutive days. Chemical or nutritional pretreatment (if any) was then instituted for four days so that the test animals were equilibrated before 3-methylcholanthrene (50 mg./kg./i.p.) or barbitone (50 mg./

rat i.p. on three successive days) was administered. Rats were given four or five days to recover from any surgical pretreatment before the treatment with 3-methylcholanthrene or barbitone.

Chemicals were given by intraperitoneal injection either as aqueous solutions (DL-ethionine, sodium barbitone), buffered solutions (SU4885 at pH 7·4), or in arachis oil (*o-p'*-DDD solution, 3-methylcholanthrene suspension). Thyroxine was administered subcutaneously. All experiments were carried out at least in duplicate.

### RESULTS

(a).—A single injection of 3-methylcholanthrene (50 mg./kg. i.p.) caused a substantial increase in ascorbic acid excretion which persisted for some weeks after a lag of two to three days (Fig. 1, curve B). Injection of barbitone (50 mg./rat i.p.) caused an immediate increase in ascorbic acid excretion which lasted for some days (Fig. 1, curve E). Rats given DL-ethionine (100 mg./kg./day i.p.) or maintained on 5 per cent protein diet for four days preceding the administration of 3-methylcholanthrene or barbitone and then continuously until the end of the experiment did not show any increase in ascorbic acid output beyond the normal range (Fig. 1, curve A). The animals so treated had a lower growth rate (*ca.* 3 g./rat/day) than the control animals (*ca.* 8 g./rat/day).

(b).—Pretreatment with compound SU4885 (100 mg./kg./day i.p.), known to reduce adrenocortical function (Brown, 1960), four days before the administration of 3-methylcholanthrene, delayed and diminished the response to 3-methylcholanthrene so that the daily output of urinary ascorbic acid was according to pattern D (Fig. 1); control values were also lowered (daily output 0·5–1·0 mg. ascorbic acid). The barbitone induced increase of ascorbic acid output is completely blocked by SU4885 (Table I).

TABLE I.—*Urinary Ascorbic Acid Excretion Patterns in Rats, as Illustrated in Fig.* 1, *Under Different Conditions*

| Pretreatment | Dose (mg./kg.) | Treatment: None | Treatment: 3-MC (50mg./kg.i.p.) | Treatment: Barbitone (50mg./rat i.p) |
|---|---|---|---|---|
| None | — | A (8) | B (6) | E (2) |
| DL-ethionine | 100 | A (2) | A (4) | A (2) |
| 5% protein diet | — | A (2) | A (2) | A (2) |
| SU4885 | 100 | A (3) | D (4) | A (2) |
| *o-p'*-DDD | 20 | C (4) | C (4) | — |
| Adrenalectomy | — | A (2) | D (2) A (4) | E (2) |
| Thyroidectomy | — | A (2) | A (3) | A (2) |
| Partial hepatectomy | — | A (4) | B (3) | — |

Figures in parentheses indicate number of animals used.

*o-p'*-DDD (20 mg./kg./day i.p.) which is an adrenal antagonist in some species (Brown, 1960) but apparently not in the rat (Hertz, 1961 personal communication), enhanced the urinary output immediately; the effect summated with that of 3-methylcholanthrene to give excretions of 20 mg. per day (Fig. 1, curve C).

A single dose of *o-p'*-DDD (100 mg./kg. i.p.) stimulated the ascorbic acid excretion more than did 3-methylcholanthrene. The effect was immediate and also of greater magnitude and duration. A daily output of more than 20 mg.

Fig. 1.—Urinary ascorbic acid excretion patterns in rats measured daily by titration with 2,6-dichlorophenol-indophenol.

A [○—○] control;

B [●—●] following an injection of 3-MC (50 mg./kg. i.p.) on day 7;

C [■—■] following daily injections of *o-p'*-DDD (20 mg./kg. i.p.) from day 3 onwards and a single injection of 3-MC (50 mg./kg. i.p.) on day 7;

D [□—□] following daily injections of SU4885 (100 mg./kg. i.p.) from day 3 onwards and a single injection of 3-MC (50 mg./kg. i.p.) on day 7;

E [△—△] following three successive injections of barbitone (50 mg./rat i.p.) on days 7, 8 and 9.

ascorbic acid was sustained for more than twenty days. The excretion thereafter declined but was still raised after thirty days.

(c).—Burns, *et al.* (1957) had found that adrenalectomy did not appear to affect the excretion of ascorbic acid in rats challenged with barbitone or chloretone. In the present experiments the ascorbic acid output in adrenalectomised rats was the same as in intact rats when the animals were given three successive doses of barbitone (Table I). However, 3-methylcholanthrene increased ascorbic acid excretion in only two out of six adrenalectomised rats (response similar to D in Fig. 1).

Thyroidectomised animals did not show any increase in urinary ascorbic acid output when 3-methylcholanthrene or barbitone were administered in the usual doses. Sham-operated animals, however, reacted like normal rats with excretion patterns B and E respectively. Thyroidectomised animals supplemented with thyroxine (0·03 mg./rat/day s.c.) for three days still behaved as thyroidectomised rats in their response to 3-methylcholanthrene and barbitone.

Partial hepatectomy was investigated in order to determine if liver regeneration caused increased synthesis of specific proteins necessary for production of intermediates in the glucuronide pathway. Partial hepatectomy itself did not change the rate of ascorbic acid output. The response of hepatectomised rats given 3-methylcholanthrene was similar to that of intact rats although the response was delayed, the peak being reached about the eleventh day after injection.

(d).—Slonaker and August rats of the same age as the Chester Beatty rats gave similar response to 3-methylcholanthrene in spite of the fact that they had a much smaller growth rate (2 g./day) and had attained weights of only 100–120 g. at the time of experimentation.

### DISCUSSION

The increase in ascorbic acid synthesis and excretion induced by carcinogens and other compounds appears to be induced by similar factors to those which increase the liver microsomal enzymes responsible for metabolising foreign compounds (Conney, Miller and Miller, 1956; Conney and Burns, 1959). Both types of effect must involve synthesis of enzymes which carry out the chemical processes involved.

The finding that DL-ethionine administration or low protein diet inhibits the response to 3-methylcholanthrene or barbitone as measured by urinary ascorbic acid output in rats, is in agreement with the concept of induced protein synthesis. However, protein synthesis *per se* does not enhance the urinary ascorbic acid output as was seen with rats that had been partially hepatectomised. Evidently the synthesis of specific liver proteins is involved in the increase of ascorbic acid excretion.

SU4885, which specifically inhibits 11–hydroxylation in $C_{19-21}$ steriods (Jenkins, Meakin, Nelson and Thorn, 1958) depressed ascorbic acid output of control and 3-methylcholanthrene stimulated rats, the effect of some 11-desoxysteroids should be investigated in connection with the phenomenon.

A surprising finding was the response elicited in rats with the other anti-adrenal compound tested, *o-p'*-DDD. *o-p'*-DDD was found to be a much more potent and long lasting stimulator of ascorbic acid output in the rat than 3-methylcholanthrene.

SUMMARY

1. The 3-methylcholanthrene and barbitone induced increases in ascorbic acid excretion in rats are dependent on dietary and hormonal factors but not on overall growth rate.

2. The induction of increased ascorbic acid output is prevented by administration of DL-ethionine or by a low protein diet.

3. Adrenalectomy sometimes neutralises the effect of 3-methylcholanthrene on ascorbic acid excretion but not that of barbitone.

4. The adrenal antagonist SU4885 suppresses the increase of ascorbic acid output induced by 3-methylcholanthrene or barbitone.

5. *o-p'*-DDD which is an anti-adrenal compound in other species enhances the induction brought about by 3-methylcholanthrene and is itself a powerful and long acting stimulator of ascorbic acid output.

6. Thyroidectomy prevents the induction of increased ascorbic acid output with 3-methylcholanthrene or barbitone.

The assistance of Mrs. S. M. A. Doak and Dr. E. J. Delorme in performing the partial hepatectomies and thyroidectomies respectively is acknowledged with thanks. We are grateful to Messrs. Ciba Laboratories Ltd. for gifts of SU4885 and to the Cancer Chemotherapy National Service Center, United States Public Health Service for a gift of *o-p'*-DDD.

This investigation has been supported by grants to the Chester Beatty Research Institute (Institute of Cancer Research: Royal Cancer Hospital) from the Medical Research Council, the British Empire Cancer Campaign, the Anna Fuller Fund, and the National Cancer Institute of the National Institutes of Health, U.S. Public Health Service.

### REFERENCES

ALLEN, M. J. AND BOYLAND, E.—(1957) *Rep. Brit. Emp. Cancer Campgn.*, **35**, 63.
BOYLAND, E. AND GROVER, P. L.—(1961) *Biochem. J.*, **81**, 163.
BROWN, J. H. U.—(1960) *Nature, Lond.*, **187**, 985.
BURNS, J. J., CONNEY, A. H., DAYTON, P. G., EVANS, C., MARTIN, G. R. and TALLER, D. —(1960) *J. Pharmacol.*, **129**, 132.
*Idem*, EVANS, C. AND TROUSOF, N.—(1957) *J. biol. Chem.*, **227**, 785.
*Idem* AND SHORE, P. A.—(1961) *Annu. Rev. Pharmacol.*, **1**, 79.
CONNEY, A. H., BRAY, G. A., EVANS, C. AND BURNS J. J.—(1961) *Ann. N.Y. Acad. Sci.*, **92**, 115.
*Idem* AND BURNS, J. J.—(1959) *Nature, Lond.*, **184**, 363.
*Idem*, MILLER, E. C. AND MILLER, J. A.—(1956) *Cancer Res.*, **16**, 450.
EVANS, C., CONNEY, A. H., TROUSOF, N. AND BURNS, J. J.—(1960) *Biochim. biophys. Acta*, **41**, 9.
HARRIS, L. J. AND OLIVER, M.—(1942) *Biochem. J.*, **36**, 155.
JENKINS, J. S., MEAKIN, J. W., NELSON, D. H. AND THORN, G. W.—(1958) *Science*, **128**, 478.
ROE, J. H. AND KUETHER, C. A.—(1943) *J. biol. Chem.*, **147**, 399.
SALOMON, L. L. AND STUBBS, D. W.—(1961) *Ann. N.Y. Acad. Sci.*, **92**, 128.
TOUSTER, O., HESTER, R. W. AND SILER, R. A.—(1960) *Biochem. biophys. Res. Commun.*, **3**, 248.
*Idem* AND HOLLMANN, S.—(1961) *Ann. N.Y. Acad. Sci.*, **92**, 318.